# Integrated Analysis of Single-Cell and Bulk RNA Sequencing Reveals HSD3B7 as a Prognostic Biomarker and Potential Therapeutic Target in ccRCC

**DOI:** 10.3390/ijms252312929

**Published:** 2024-12-01

**Authors:** Guicen Liu, Qichen Liu, Jiawei Zhao, Ruyue Luo, Yuan Wan, Zhongli Luo

**Affiliations:** 1 Molecular Medicine and Cancer Research Center, School of Basic Medical Science, Chongqing Medical University, 400016 Chongqing, China; 2 College of Paediatrics, Chongqing Medical University, 400016 Chongqing, China

**Keywords:** scRNA-seq, bulk RNA-seq, HSD3B7, ccRCC

## Abstract

Clear cell renal cell carcinoma (ccRCC) is the most common kidney malignancy, with a poor prognosis for advanced-stage patients. Identifying key biomarkers involved in tumor progression is crucial for improving treatment outcomes. In this study, we employed an integrated approach combining single-cell RNA sequencing (scRNA-seq) and bulk RNA sequencing (bulk RNA-seq) to identify biomarkers associated with ccRCC progression and prognosis. Single-cell transcriptomic data were obtained from publicly available datasets, and genes related to tumor progression were screened using Monocle2. Bulk RNA-seq data for ccRCC were retrieved from The Cancer Genome Atlas (TCGA) and integrated with scRNA-seq data to explore tumor heterogeneity. We identified 3 beta-hydroxy steroid dehydrogenase type 7 (HSD3B7) as a candidate biomarker for ccRCC, associated with poor overall survival, disease-specific survival, and progression-free interval. Elevated HSD3B7 expression correlated with aggressive clinical features such as advanced TNM stages, histologic grades, and metastasis. Functional studies demonstrated that HSD3B7 promotes cell proliferation, migration, and invasion in vitro, while its silencing significantly inhibits tumor growth in vivo. Our findings reveal that HSD3B7 is a novel biomarker for ccRCC, providing insights into its role in tumor progression and potential as a target for therapy. This study highlights the value of integrating scRNA-seq and bulk RNA-seq data to uncover key regulators of tumor biology and lays the foundation for developing personalized therapeutic strategies for ccRCC patients.

## 1. Introduction

The incidence of kidney cancer is increasing by approximately 1.5% annually [1]. Renal cell carcinoma (RCC), the most common form of kidney cancer, can be classified into several major subtypes: clear cell RCC (ccRCC), papillary RCC (PRCC), chromophobe RCC (ChRCC), and oncocytoma [2]. ccRCC, which originates from the epithelial cells of the proximal tubules in the kidney and accounts for approximately 75% of RCC cases, is characterized by von Hippel–Lindau tumor suppressor (VHL) gene mutations, high angiogenesis, and significant metabolic reprogramming, making it the most aggressive subtype [3]. PRCC, the second most common type, is often linked to MET proto-oncogene, receptor tyrosine kinase (MET) gene alterations, while ChRCC exhibits multiple chromosomal losses and generally has a better prognosis. Oncocytoma, typically benign, is characterized by unique mitochondrial features [2]. ccRCC is responsible for most of the deaths associated with RCC [4]. Despite advances in diagnostic and treatment methods, the prognosis for patients remains poor due to the high heterogeneity and aggressiveness of the disease, with a 5-year survival rate of less than 10% for those with advanced stages [5,6,7,8]. Current treatment options for ccRCC include surgery, radiotherapy, and targeted therapies [9]. Commonly used targeted drugs for ccRCC include tyrosine kinase inhibitors (TKIs), mechanistic target of rapamycin kinase (mTOR) inhibitors, immune checkpoint inhibitors, and hypoxia-inducible factor-2α inhibitors [10,11,12]. However, treatment efficacy remains constrained by drug resistance, adverse side effects, and variability in patient response. Therefore, exploring new treatment strategies to improve patient prognosis is urgently needed.

Tumor driver genes are those that play a role in tumor initiation and progression by promoting cell proliferation, survival, and metastasis [13]. Numerous studies have shown that the occurrence and progression of ccRCC are associated with extensive genetic alterations, especially mutations, deletions, and amplifications of key oncogenes and tumor suppressor genes [14,15,16,17,18]. Several common mutated genes in ccRCC have been identified, including VHL, polybromo 1 (PBRM1), SET domain containing 2, histone lysine methyltransferase (SETD2), phosphatase and tensin homolog (PTEN), and tumor protein p53 (TP53) [19,20,21,22,23]. These genes play various roles in the progression of ccRCC, including regulating cell proliferation, angiogenesis, and metabolism [24,25]. Targeting these driver genes has shown clinical efficacy in ccRCC treatment [26]. The identification of driver genes in ccRCC is crucial for developing effective targeted therapies and personalized treatment strategies. Given the extensive genetic alterations involved in ccRCC, identifying driver genes is a complex and challenging task.

Single-cell RNA sequencing (scRNA-seq) is an advanced technique that enables the exploration of transcriptomic heterogeneity at the single-cell level [27,28,29]. Unlike bulk RNA sequencing (bulk RNA-seq), which measures averaged gene expression across heterogeneous cell populations, scRNA-seq identifies distinct cell types, subpopulations, and their specific gene expression [30,31,32]. This method has been instrumental in revealing cellular diversity and the complex interactions between malignant and non-malignant cells in various cancers, including ccRCC. Integrating scRNA-seq with bulk RNA-seq offers a comprehensive understanding of tumor heterogeneity and identifies key molecular markers associated with disease progression. Recent studies emphasize the importance of multi-omics data integration to elucidate the molecular mechanisms underlying tumorigenesis and progression, potentially guiding the development of personalized therapeutic strategies [33,34,35,36,37].

In this study, we conducted an integrated analysis of single-cell and bulk RNA-seq data from publicly available datasets, including The Cancer Genome Atlas (TCGA) and Gene Expression Omnibus (GEO). Our goal was to identify biomarkers associated with tumor progression and prognosis in ccRCC by leveraging both single-cell and bulk transcriptomic data. These findings contribute to a deeper understanding of ccRCC biology and provide a reference for future research into personalized treatment approaches for ccRCC patients.

## 2. Results

### 2.1. Establishment of a Single-Cell Atlas for ccRCC

**Figure 1:**
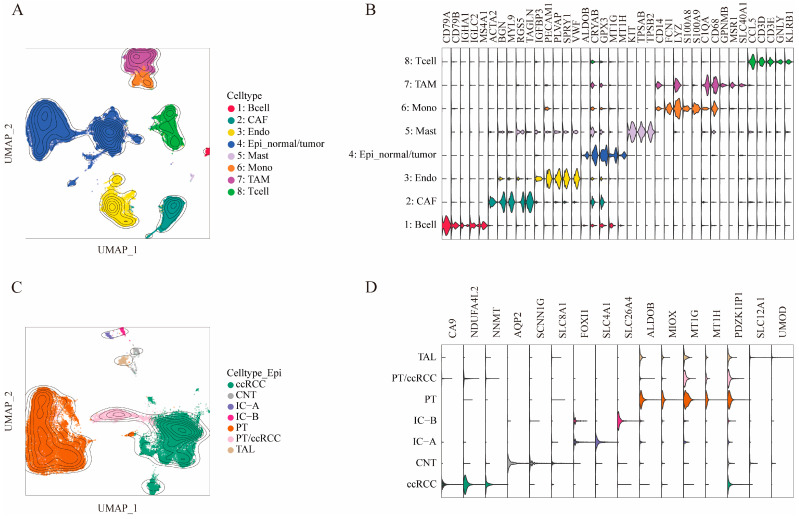
Single-cell transcriptomic atlas following the integration of multiple datasets. (**A**,**C**) Kernel density plots illustrating cell type clustering across the integrated datasets, where each point represents a single cell, and each color corresponds to a specific cell type. Each contour line represents areas of different cell densities, with closer lines indicating higher cell densities in that region. (**B**,**D**) Violin plots displaying the expression levels of specific marker genes for each cell type, where violins of the same color represent marker genes for the same cell type.

After quality control filtering, 96,175 cells were collected from 23 samples for downstream analyses. Cell type identities were assigned using cell type-specific markers previously reported in the literature (Figure 1B,D). The cells were categorized into eight main types based on gene expression profiles (Figure 1A): B cells (Bcell; *n* = 613), cancer-associated fibroblasts (CAF; *n* = 8209), endothelial cells (Endo; *n* = 12,621), epithelial cells (Epi_normal/tumor; *n* = 48,298), mast cells (Mast; *n* = 272), monocytes (Mono; *n* = 4031), tumor-associated macrophages (TAM; *n* = 11,241), and T cells (Tcell; *n* = 9460). In total, 1430 cells were recognized as doublets and subsequently removed. Additionally, epithelial subclusters were identified and are shown in Figure 1C, representing distinct cell types: proximal tubular cells (PT; *n* = 22,985), tumor-specific cells (ccRCC, *n* = 15,927), PT/ccRCC (*n* = 2607), connecting tubule cells (CNT, *n* = 436), thick ascending limb cells (TAL, *n* = 509), and collecting duct cells (IC-A, *n* = 307; IC-B, *n* = 286). In total, 5241 cells were identified as doublets and removed. The details regarding the origin of the original datasets are provided in Table S1.

### 2.2. Pseudotime Differential Gene Screening

**Figure 2:**
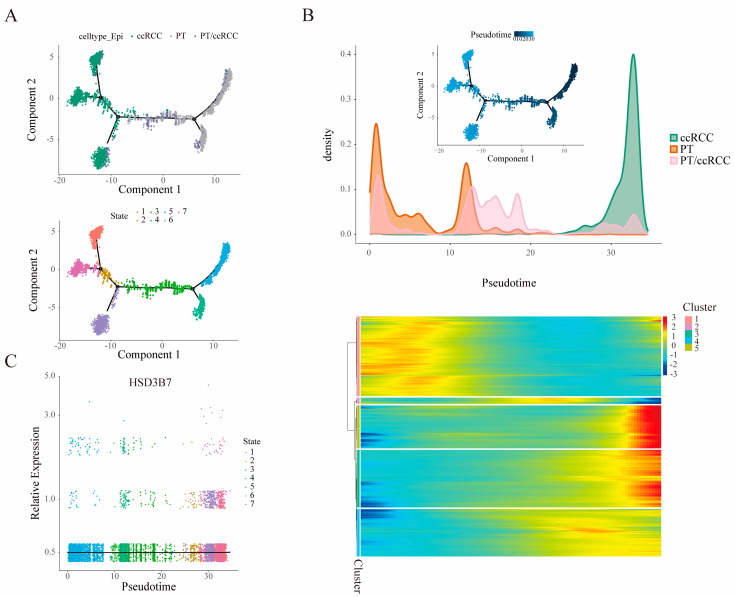
Identification of HSD3B7. (**A**) Developmental trajectory of epithelial cells based on Monocle2 analysis. Each dot represents a cell, and the lines represent differentiation trajectories. (**B**) The upper panel displays the distribution of epithelial cells along pseudotime. Heatmap of differentially expressed genes, ordered according to their common expression variation through pseudotime (gene sets 1–5). (**C**) Expression pattern of the HSD3B7 gene along the pseudotime.

Figure 2 presents the trajectory analysis of epithelial cells in ccRCC using Monocle2 pseudotime analysis. Tumor cells were primarily distributed in states 1, 2, 6, and 7 (Figure 2A). Figure 2B shows the density distribution of different epithelial cell types, including ccRCC, PT, and a mixed state of PT and ccRCC cells across pseudotime, corresponding to the annotated results of epithelial cell subpopulation analysis. By setting “num_clusters = 5”, pseudotime differential genes were clustered into five groups. Genes from cluster 5 were extracted and integrated with bulk RNA-seq data, identifying 3 beta-hydroxy steroid dehydrogenase type 7 (HSD3B7) as a critical gene involved in tumorigenesis for further research. Figure 2C displays the relative expression of HSD3B7, with higher levels observed at later pseudotime points, suggesting a potential role in tumor progression.

### 2.3. HSD3B7 as a Novel Biomarker for Prognosis and Clinical Features

**Figure 3:**
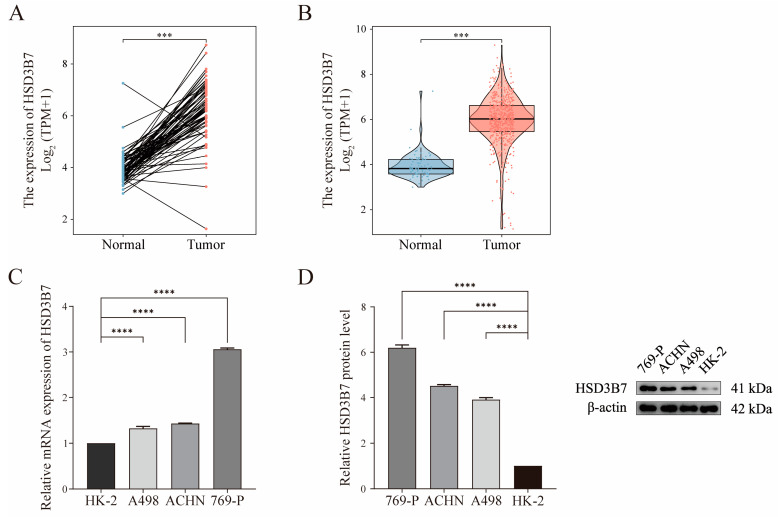
Upregulation of HSD3B7 in ccRCC. Differential expression of HSD3B7 in paired (**A**) and unpaired (**B**) normal adjacent and tumor samples from TCGA-KIRC dataset. Each dot represents a sample. Relative expression of HSD3B7 in ccRCC cells compared to normal kidney cells was determined by quantitative real-time PCR (qRT-PCR) (**C**) and Western blot (WB) (**D**). *** *p* < 0.001, **** *p* < 0.0001.

**Figure 4:**
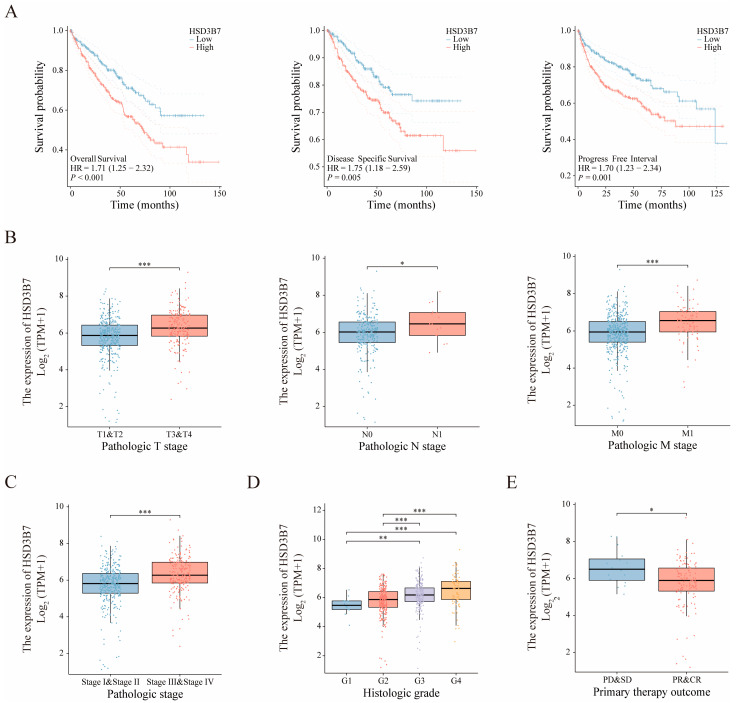
Association of HSD3B7 expression with survival outcomes and clinical features. (**A**) Kaplan–Meier curves comparing OS, DSS, and PFI between patients with high and low HSD3B7 expression. (**B**–**E**) Expression levels of HSD3B7 across various pathological conditions and primary therapy outcomes. * *p* < 0.05, ** *p* < 0.01, *** *p* < 0.001.

HSD3B7 expression was significantly higher in ccRCC compared to adjacent normal samples, as indicated by both mRNA and protein levels (Figure 3). In the integrated multi-dataset UMAP analysis, HSD3B7 showed the highest expression in epithelial cells (Figure S1A). Further subtype analysis of epithelial cells revealed that HSD3B7 expression was elevated specifically in tumor epithelial cells compared to normal epithelial cells, suggesting its potential role in tumor progression (Figure S1B). Figure 4A shows that patients with high HSD3B7 expression had significantly poorer overall survival (OS; HR = 1.71, 95% CI: 1.25–2.32, *p* < 0.001), disease-specific survival (DSS; HR = 1.75, 95% CI: 1.18–2.59, *p* = 0.005), and progress free interval (PFI; HR = 1.70, 95% CI: 1.23–2.34, *p* = 0.001). Elevated HSD3B7 expression was associated with advanced pathological T stages (T3 and T4 vs. T1 and T2), N stages (N1 vs. N0), M stages (M1 vs. M0), higher histologic grades (G3 and G4 vs. G1 and G2), and more advanced disease stages (Stage III and IV vs. Stage I and II) (Figure 4B–D). Patients with progressive or stable disease (PD/SD) also exhibited higher HSD3B7 expression compared to those with partial or complete response (PR/CR) (Figure 4E). These findings suggest that HSD3B7 expression correlates with more aggressive tumor characteristics and poorer clinical outcomes.

### 2.4. Elevated HSD3B7 Expression Enhances the Progression of ccRCC

**Figure 5:**
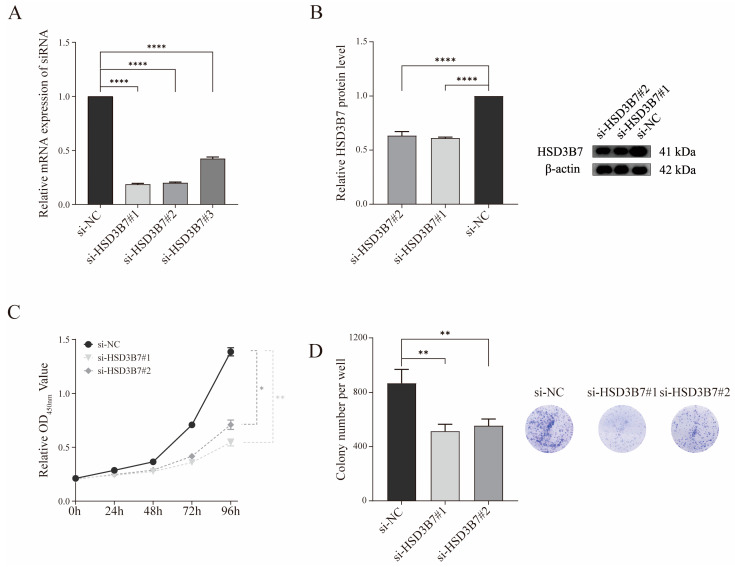
Effects of HSD3B7 knockdown on cell proliferation. (**A**) qRT-PCR indicated a knockdown efficiency exceeding 50% in 769-P cells. (**B**) WB revealed the knockdown efficiency of HSD3B7 in 769-P cells. (**C**) The proliferative activity of cells was measured using the CCK-8 assay following knockdown of HSD3B7 expression. (**D**) The impact of HSD3B7 on the proliferative capacity of 769-P cells was evaluated using a colony formation assay. ** *p* < 0.01, **** *p* < 0.0001.

The efficiency and duration of HSD3B7 knockdown at the mRNA level were validated in 769-P cells over a 96 h period (Figure S2). qRT-PCR analysis and WB showed that HSD3B7 mRNA and protein levels were effectively knocked down in cells transfected with three different HSD3B7-targeting small interfering RNAs (siRNAs) compared to the negative control (Figure 5A,B). The Cell Counting Kit-8 (CCK-8) assay revealed a significant reduction in cell proliferation in HSD3B7-knockdown cells compared to controls over 96 h (Figure 5C). The colony formation assay demonstrated a significant decrease in the number of colonies per well in HSD3B7-knockdown cells (Figure 5D), indicating suppressed proliferative capacity.

**Figure 6:**
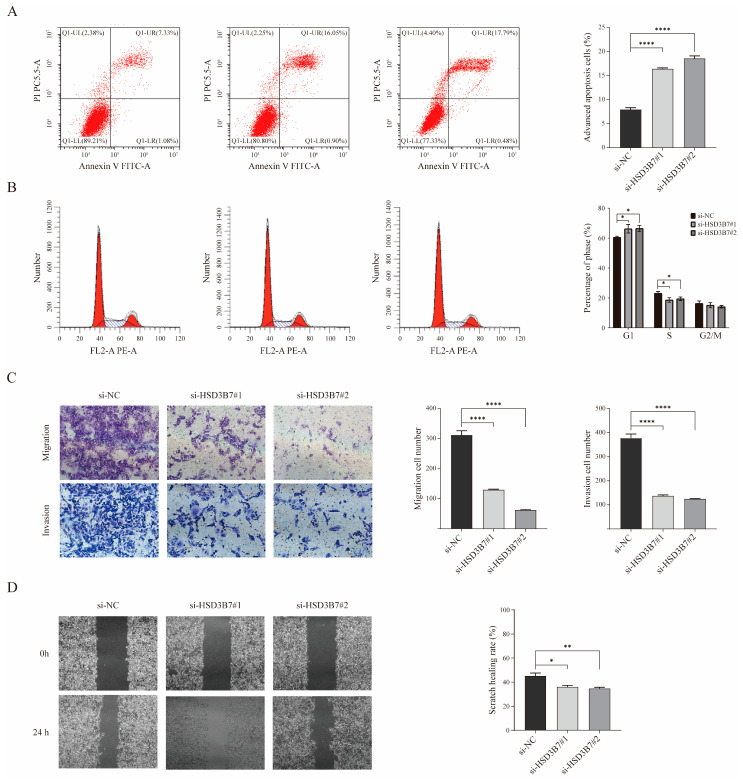
Impact of HSD3B7 knockdown on apoptosis, cell cycle, migration, and invasion. (**A**,**B**) Apoptosis and cell cycle alterations in ccRCC cells following HSD3B7 knockdown were assessed using flow cytometry. (**C**) The migratory and invasive capabilities of 769-P cells under varying transfection conditions were analyzed via Transwell assay. (**D**) The cell scratch assay revealed a significant decrease in migration ability in the HSD3B7 knockdown group compared to the control group. * *p* < 0.05, ** *p* < 0.01, **** *p* < 0.0001.

Flow cytometry analysis showed a significant increase in the percentage of apoptotic cells in the HSD3B7-knockdown groups (Figure 6A). Cell cycle analysis demonstrated that HSD3B7 knockdown led to an increased percentage of cells in the G1 phase and a decreased percentage in the S phase, indicating cell cycle arrest at the G1 phase (Figure 6B). Transwell migration and invasion assays showed a significant reduction in the number of migrated and invading cells in the HSD3B7-knockdown groups, suggesting impaired migratory and invasive abilities (Figure 6C). The wound healing assay revealed a lower scratch healing rate in cells transfected with si-HSD3B7#1 and si-HSD3B7#2, indicating reduced cellular migration and wound healing capacity (Figure 6D). These results indicate that HSD3B7 knockdown increases apoptosis, induces cell cycle arrest, and reduces migration and invasion capabilities.

**Figure 7:**
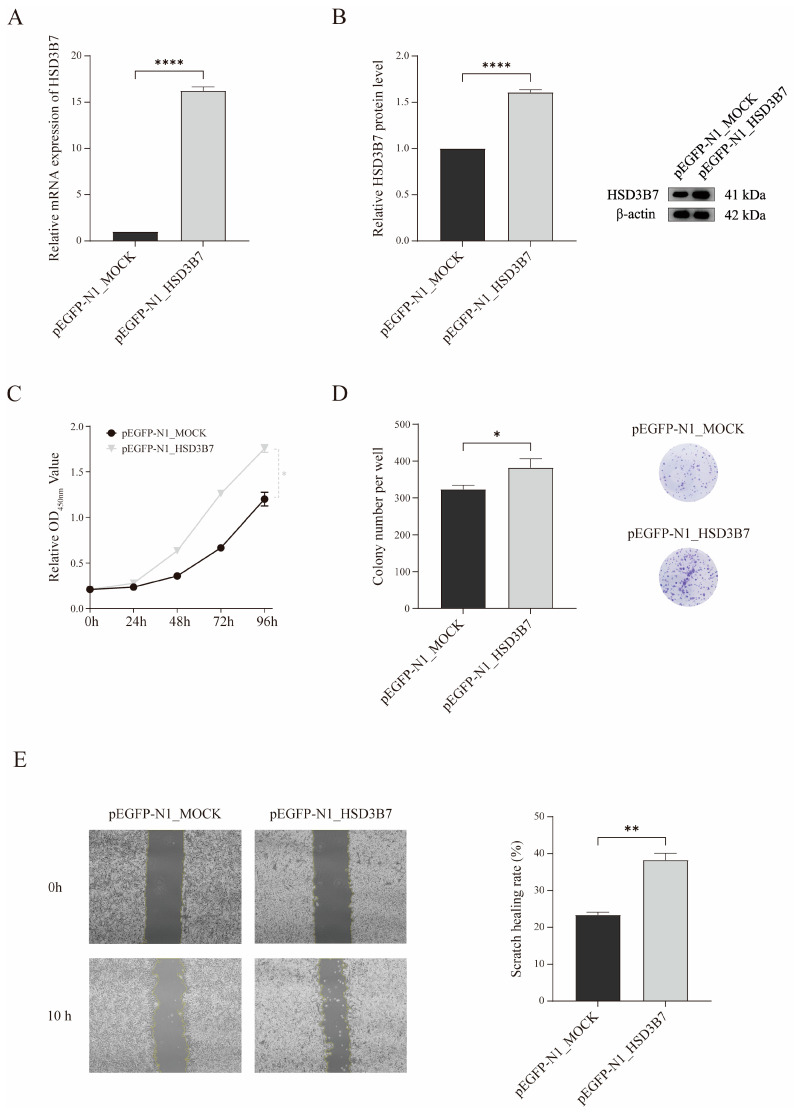
Effects of HSD3B7 overexpression on renal carcinoma cell proliferation, colony formation, and migration. (**A**,**B**) The relative expression of HSD3B7 in A498 cells transfected with the indicated vectors was assessed using qRT-PCR and WB. (**C**) The proliferative activity of the cells following HSD3B7 overexpression was measured using the CCK-8 assay. (**D**) The impact of HSD3B7 on the proliferative capacity of A498 cells was evaluated using a colony formation assay. (**E**) The effect of HSD3B7 on the migratory capacity of A498 cells was assessed using a wound healing assay. * *p* < 0.05, ** *p* < 0.01, **** *p* < 0.0001.

qRT-PCR and WB analysis indicated that HSD3B7 levels were significantly elevated in the overexpression group compared to the mock vector group (Figure 7A,B). The CCK-8 assay showed enhanced cell proliferation in HSD3B7-overexpressing cells compared to controls at multiple time points (0, 24, 48, 72, and 96 h) (Figure 7C). The colony formation assay demonstrated an increased number of colonies per well in HSD3B7-overexpressing cells, indicating enhanced long-term proliferative capacity (Figure 7D). The wound healing assay showed a significantly higher scratch healing rate in HSD3B7-overexpressing cells compared to controls, suggesting that HSD3B7 promotes cellular migration and wound healing capabilities (Figure 7E). These findings collectively indicate that HSD3B7 overexpression in A498 renal carcinoma cells enhances proliferation, colony formation, and migration.

### 2.5. In Vivo Silencing of HSD3B7 Reduces Tumor Growth

**Figure 8:**
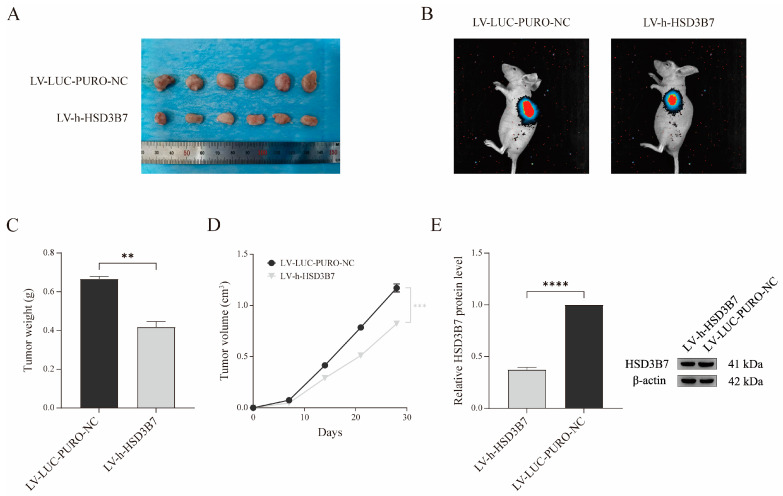
Effects of HSD3B7 silencing on tumor growth in a xenograft model using nude mice. (**A**) Representative images of nude mice in each group (*n* = 6). (**B**) Representative bioluminescence images of the subcutaneous tumor model in each group of nude mice. (**C**) Comparison of tumor weights across groups (*n* = 6). (**D**) Weekly measurements of tumor xenograft growth curves for nude mice in each group (*n* = 6). (**E**) WB analysis of HSD3B7 protein levels in tumor tissues from the nude mouse xenograft model following silencing. ** *p* < 0.01, **** *p* < 0.0001.

To investigate the role of HSD3B7 in vivo, 769-P cells with HSD3B7 knockdown were subcutaneously injected into nude mice to establish a xenograft model. Tumor growth was monitored for four weeks. Mice injected with HSD3B7-silenced cells developed significantly smaller tumors (Figure 8A,B). Tumor weight, measured at the end of the 30-day observation period, was significantly lower in the LV-h-HSD3B7 group compared to controls, suggesting that HSD3B7 silencing inhibits tumor growth in vivo (Figure 8C). Tumor volume was measured every seven days over a 28-day period, showing that tumors derived from HSD3B7-silenced cells grew significantly more slowly than those in the control group (Figure 8D). Additionally, Figure 8E presents the results of WB analysis of HSD3B7 protein levels in tumor tissues from these mice, confirming that HSD3B7 expression was significantly reduced in the HSD3B7-silenced tumors compared to the control group. These findings indicate that HSD3B7 silencing significantly reduces tumorigenic potential in renal carcinoma xenograft models, supporting its role in promoting tumor growth and suggesting its potential as a therapeutic target in ccRCC.

## 3. Discussion

In this study, we performed an integrated analysis of single-cell and bulk RNA-seq data to identify biomarkers associated with tumor progression and prognosis in ccRCC. By combining scRNA-seq and bulk RNA-seq approaches, we aimed to capture both the specific cell subpopulations within tumors and the broader tumor-level heterogeneity, thereby providing a comprehensive understanding of ccRCC. Our trajectory analysis revealed that HSD3B7 expression was upregulated in the later stages of pseudotime, suggesting its involvement in tumor progression. This was further corroborated by bulk RNA-seq data, which confirmed the potential of HSD3B7 as a candidate target gene.

Our findings identified HSD3B7 as a critical gene involved in ccRCC progression. Elevated HSD3B7 expression was significantly correlated with poorer clinical outcomes, including OS, DSS, and PFI, suggesting its potential as a prognostic biomarker in ccRCC. Furthermore, in vitro and in vivo functional studies demonstrated that HSD3B7 overexpression enhances cell proliferation, migration, and invasion, while its silencing suppresses these oncogenic properties. These results align with previous findings in other malignancies, indicating that HSD3B7 plays a crucial role in promoting cancer aggressiveness [38,39,40].

HSD3B7 is involved in steroid metabolism, and its role in cancer biology is increasingly recognized [41,42,43]. Riscal et al. focused on the metabolic role of HSD3B7 in cholesterol metabolism, investigating its impact on tumorigenesis through both in vitro and in vivo experiments targeting metabolic vulnerabilities [43]. Their study demonstrated that inhibition of HSD3B7 led to the accumulation of toxic oxysterols, which induced apoptosis in ccRCC cells. This highlighted the therapeutic potential of targeting metabolic pathways involving HSD3B7 in ccRCC. However, Riscal et al.’s selection of HSD3B7 was primarily driven by its role in cholesterol homeostasis, without exploring its broader impact on tumor progression and heterogeneity. Another study by Hu et al. primarily utilized bioinformatics analyses to explore correlations between HSD3B7 and immune cell infiltration, DNA methylation, and prognosis in ccRCC [44]. This study relied solely on computational data to establish HSD3B7 as a prognostic biomarker, but the lack of experimental validation limited understanding of its functional role and underlying mechanisms in ccRCC progression.

While both studies provided significant contributions to understanding the role of HSD3B7 in ccRCC, our study offers a more comprehensive perspective by integrating multiple methodologies. We employed a systems biology approach for a holistic screening of target genes, which allowed us to identify critical biomarkers associated with tumor progression and prognosis. This method ensured that candidate genes were selected based on a broader understanding of the biological system rather than isolated pathways, providing a robust foundation for downstream validation.

Moreover, we conducted both in vitro and in vivo validations to confirm the functional role of HSD3B7 in ccRCC progression. The experimental validation demonstrated that HSD3B7 overexpression promotes cell proliferation, migration, and invasion, while silencing HSD3B7 inhibits these oncogenic behaviors. Additionally, we analyzed its association with survival and prognosis, providing a comprehensive understanding of its clinical value and biological significance. This combination of experimental validation and clinical correlation enhances the reliability and translational potential of our findings.

It is also important to acknowledge that scientific conclusions require validation across multiple independent studies [45,46,47]. By incorporating multiple levels of experimental validation and corroborating our findings with existing literature, we provide a strong basis for the role of HSD3B7 in ccRCC, emphasizing its potential as both a prognostic marker and a therapeutic target. This comprehensive and iterative validation approach strengthens the overall impact and novelty of our research.

The implications of our findings extend beyond the identification of a novel biomarker. By integrating single-cell and bulk RNA-seq, we provided a comprehensive understanding of the molecular mechanisms underlying ccRCC progression. This multi-omics integration underscores the importance of a holistic approach to identifying actionable targets, which could pave the way for personalized treatment strategies that address tumor heterogeneity.

Nevertheless, our study has several limitations. Although we utilized both publicly available datasets and experimental validation to identify and characterize HSD3B7, further investigations are needed to fully elucidate the molecular mechanisms underlying its oncogenic activity in ccRCC. In our study, we utilized the 769-P cell line for siRNA-mediated knockdown of HSD3B7 and the A498 cell line for HSD3B7 overexpression experiments due to the expression level of HSD3B7. These cell lines are well-characterized and commonly used in ccRCC research, but expanding the study to include a broader range of ccRCC cell lines for both knockdown and overexpression experiments would provide a more comprehensive understanding of the diverse roles of HSD3B7 in ccRCC progression. Moreover, while in vivo models provide valuable insights into tumor behavior, they may not fully capture the complexity of human tumors. Therefore, increasing the clinical sample size and validating HSD3B7 as a therapeutic target in patient-derived xenografts and clinical specimens are essential next steps. While the findings from this study are promising, further validation is necessary to strengthen these conclusions and confirm the translational potential of HSD3B7 as a therapeutic target.

## 4. Materials and Methods

### 4.1. Data Acquisition

scRNA-seq datasets (GSE131685, GSE152938, GSE156632, and GSE159115) were obtained from the Gene Expression Omnibus (GEO; https://www.ncbi.nlm.nih.gov/geo, accessed on 2 November 2023). Data from GSE131685 provided transcriptomic profiles of 23,366 cells from three normal kidney samples. GSE152938 included scRNA-seq data of 20,599 cells from two ccRCC tumor tissues. GSE156632 analyzed 54,221 cells from six ccRCC tumor samples and five adjacent normal samples. GSE159115 generated a comprehensive cell atlas of 26,180 cells from three benign and seven tumor samples. Samples GSM4819726, GSM4819728, and GSM4819735 were excluded due to high mitochondrial gene content. All scRNA-seq datasets were generated using the 10X Genomics platform. Bulk RNA-seq and clinical data for ccRCC were retrieved from the Genomic Data Commons (GDC) data portal (https://portal.gdc.cancer.gov/, accessed on 24 November 2023). After excluding samples without clinical information and duplicates, 532 tumor samples and 72 adjacent normal samples were retained, including 72 paired samples.

### 4.2. Processing and Quality Control of Single-Cell Datasets

For scRNA-seq data, initial processing was conducted using the Cell Ranger pipeline (10X Genomics, CA, USA), followed by quality control with the Seurat R package (v4.3.2; https://www.R-project.org, accessed on 2 November 2023) [48,49]. Cells were retained based on the following criteria: retention of features between 300 and 5000, unique molecular identifier (UMI) counts between 1000 and 20,000, genes expressed in 10 or more cells, and a mitochondrial UMI proportion below 15%. Doublets identified by DoubletFinder (v2.0.4) were excluded, with an 8% doublet expectation [50]. Sequencing depth normalization was performed using the “SCTransform” function and the “glmGamPoi” method. The 3000 most variable features were identified using the “vst” method, and significant principal components were detected using principal component analysis (PCA). Data integration was performed with RunHarmony implemented in Seurat [51]. Cell clustering analysis was conducted using the FindNeighbors and FindClusters functions. Uniform Manifold Approximation and Projection (UMAP) analysis was subsequently performed using the RunUMAP in Seurat. Principal components were selected based on the following criteria: cumulative contribution >90%, individual component variance contribution <5%, and the difference between two consecutive components <0.1%.

To predict cellular differentiation, cells were ordered in pseudotime using Monocle2 (v2.26.0) [52]. Differential gene expression along the cell trajectory was assessed using differentialGeneTest (qval < 0.01). Cell trajectory, gene expression across pseudotime, and a heatmap of pseudotime plots were generated using plot_cell_trajectory, plot_genes_in_pseudotime (min_expr = 0.1, trend_formula = “~ sm.ns (Pseudotime, df = 5)”), and plot_pseuodtime_heatmap, respectively.

### 4.3. Processing of Bulk RNA-seq Data

Bulk RNA-seq data were processed using the STAR aligner, and differential gene expression analysis between tumor and normal tissues was performed using the “stats” (v4.2.1) and “car” (v3.1-0) packages [53,54,55]. The expression data were transformed to log2 [transcripts per million (TPM) + 1]. Duplicated samples for each case were excluded. To investigate the relationship between gene expression and patient prognosis, three outcome measures were selected: OS, DSS, and PFI. Gene expression was categorized into low and high groups based on the median. Univariate Cox regression analysis was performed using the R package “survival” (v3.3.1, https://cran.r-project.org/web/packages/survival/index.html, accessed on 24 November 2023) and “survminer” (v0.4.9, https://cran.r-project.org/web/packages/survminer/index.html, accessed on 24 November 2023) to evaluate and visualize the association between patient survival and gene expression. The Kruskal–Wallis test or Wilcoxon rank-sum test was used to analyze the differences in gene expression among tumor stages, pathologic stage, histologic grade, and primary therapy outcome. All plots were generated using “ggplot2” (v3.3.6) [56].

### 4.4. Cell Culture and Transfection

All cell lines used in this study, including A498, ACHN, 769-P, and HK-2, are adherent cells purchased from Procell (Wuhan, China), and all experiments were conducted using cells confirmed to be free of mycoplasma contamination. A498 and 769-P were derived from female RCC patients, while ACHN was established from a male patient’s pleural metastasis. All three tumor cell lines (A498, ACHN, and 769-P) are well-established models for studying ccRCC, whereas HK-2, derived from normal human proximal tubular epithelial cells, serves as a non-cancerous control. To assess the functional role of HSD3B7 in ccRCC, in vitro experiments were conducted using these cell lines. A498, ACHN, and HK-2 cells were cultured in Dulbecco’s Modified Eagle Medium (DMEM, Gibco, CA, USA) supplemented with 10% fetal bovine serum (FBS, Procell, Wuhan, China) and 1% penicillin–streptomycin (Gibco, MA, USA), while 769-P cells were cultured in RPMI-1640 medium (Gibco, MA, USA) containing 10% FBS and 1% penicillin–streptomycin. All cell lines were maintained in a humidified incubator at 37 °C with 5% CO_2_.

For knockdown experiments, siRNA targeting HSD3B7 was transfected into 769-P cells using Lipofectamine 3000 (Thermo Fisher Scientific, Cleveland, OH, USA) following the manufacturer’s protocol. A non-targeting siRNA served as the control. The siRNA sequences used in the study are listed in Table S2. For overexpression studies, HSD3B7 was cloned into a pcDNA3.1 vector, and the overexpression plasmids were transfected into A498 cells using the same transfection protocol. The siRNAs and HSD3B7 overexpression plasmids were provided by Sangon Bioengineering (Shanghai, China).

### 4.5. qRT-PCR

Total RNA was extracted from cells using Trizol reagent (Tiangen, Beijing, China) and assessed by Nanodrop and agarose gel electrophoresis. RNA was then reverse-transcribed into cDNA using the PrimeScript RT reagent kit (Takara, Dalian, China). TB Green Premix Ex Taq (Takara, Shiga, Japan) was used to amplify the cDNA on a Bio-Rad CFX96 system (Bio-Rad, Hercules, CA, USA). The primers used in the study are listed in Table S3.

### 4.6. WB Analysis

Total protein was extracted from cells using radioimmunoprecipitation assay (RIPA; Beyotime, Shanghai, China) lysis buffer containing phenylmethylsulfonyl fluoride (PMSF; Keygentec, Nanjing, China). The lysates were sonicated on ice and centrifuged at 12,000 rpm for 5 min at 4 °C to remove debris. Protein concentrations were determined using the bicinchoninic acid (BCA, Keygentec, Nanjing, China) assay following the manufacturer’s instructions. Equal amounts of protein (20 µg per sample) were separated by SDS-PAGE and transferred onto polyvinylidene difluoride (PVDF) membranes (Millipore, Billerica, MA, USA) using a wet transfer system. Membranes were blocked with blocking buffer for WB (Beyotime, Haimen, China) for 1 h at room temperature and incubated with primary antibodies overnight at 4 °C. The primary antibodies used were HSD3B7 (1:1000, Abcam, Cambridge, UK) and β-actin (1:5000, Proteintech, Wuhan, China). After washing with TBST, membranes were incubated with a secondary antibody (1:5000, Proteintech, Rosemont, IL, USA) for 1.5 h at room temperature. Protein bands were visualized using the SuperSignal West Atto (Thermo Fisher Scientific, Waltham, MA, USA), and band intensity was quantified using image analysis software. Protein levels were normalized to the internal control, β-actin.

### 4.7. Cell Proliferation and Clonogenic Assay

Cell proliferation was evaluated using the CCK-8 (DOJINDO, Kumamoto, Japan) assay. Transfected cells were seeded in 96-well plates, and absorbance at 450 nm was measured at multiple time points. For the clonogenic assay, transfected cells (2000 cells per well) were seeded into 6-well plates and cultured for two weeks. After the culture period, cells were fixed and stained to visualize and count colonies, defined as clusters containing at least 50 cells.

### 4.8. Flow Cytometry for Apoptosis and Cell Cycle Analysis

For apoptosis analysis, transfected cells were stained with the Annexin V-FITC/PI Apoptosis Detection Kit (Beyotime, Shanghai, China) and analyzed by flow cytometry to determine the percentage of apoptotic cells. For cell cycle analysis, transfected cells were fixed in ethanol, stained with propidium iodide, and analyzed by flow cytometry to assess cell cycle distribution.

### 4.9. Cell Migration and Invasion Assay

For Transwell migration and invasion assays, transfected cells were seeded in the upper chamber of an 8.0 µm pore size Transwell insert (Corning, New York, NY, USA), and migration or invasion was assessed after incubation. For invasion assays, the upper chamber was coated with Matrigel (Corning, New York, NY, USA), and non-invading cells were removed before analysis.

Migration was also assessed using a wound-healing assay, where confluent transfected cells were scratched, washed, and cultured in serum-free medium. Images were captured at different time points to evaluate wound closure.

### 4.10. Model Animal Experiments

Female BALB/c nude mice (4 weeks old) were purchased from Hunan SJA Laboratory Animal Co., Ltd. (Changsha, China) and housed under standard conditions at the Experimental Animal Center of Chongqing Medical University. The mice were acclimated for two weeks under specific pathogen-free (SPF) conditions with a 12 h light/dark cycle and provided with ad libitum access to food and water. To assess tumorigenic potential, 769-P cells with stable HSD3B7 silencing were harvested and resuspended in 100 µL of ice-cold Matrigel at a concentration of 2 × 10^7^ cells/mL. Cells were carefully mixed with Matrigel to ensure homogeneous distribution before injection. The prepared cell suspension was then subcutaneously injected into the dorsal skin of 6-week-old female BALB/c nude mice. Tumor growth was monitored weekly, beginning at the point of visible tumor formation, using digital calipers to measure both the length and width of the developing tumors. Measurements were taken by two independent researchers to ensure consistency and minimize observer bias. Tumor volume was calculated using the formula: (length × width^2^)/2, where the length was defined as the longest dimension and the width was the perpendicular measurement. After 4 weeks post-injection, mice were administered D-luciferin potassium salt, which was prepared by dissolving in D-PBS to a final concentration of 15 mg/mL and filtered through a 0.22 μm membrane for sterilization. The freshly prepared solution was injected intraperitoneally at a dose of 10 μL/g body weight. Following administration, tumors were imaged using a small animal imaging system (Berthold, Wildbad, Germany) to monitor tumor growth and luciferase activity. After imaging, the mice were euthanized to excise the tumors, which were subsequently weighed and photographed. A portion of the excised tumor was immediately used for total protein extraction, while the remaining tissue was fixed in 4% paraformaldehyde for further histological analysis. All animal procedures were approved by the Ethics Committee of Chongqing Medical University.

### 4.11. Statistical Analysis

All in vitro and in vivo experiments were performed in triplicate, and data are presented as mean ± standard error of the mean (SEM) using GraphPad Prism 10 (GraphPad, prism software, v10.2). Statistical significance was determined using Student’s *t*-test for comparisons between two groups and one-way analysis of variance (ANOVA) followed by Tukey’s post-hoc test for multiple comparisons. A *p*-value < 0.05 was considered statistically significant.

## 5. Conclusions

In conclusion, our study identified HSD3B7 as a novel prognostic biomarker and potential therapeutic target in ccRCC through an integrated analysis of single-cell and bulk RNA-seq data. By elucidating the role of HSD3B7 in tumor progression, we provide new insights into the molecular mechanisms of ccRCC. These findings highlight the importance of HSD3B7 in ccRCC progression and offer a foundation for developing more effective, personalized therapeutic strategies for ccRCC patients.

## Data Availability

Publicly available datasets were analyzed in this study. scRNA-seq datasets (GSE131685, GSE152938, GSE156632, and GSE159115) were obtained from the Gene Expression Omnibus (GEO; https://www.ncbi.nlm.nih.gov/geo (accessed on 28 October 2024)). The ccRCC bulk RNA-seq and clinical data were retrieved from the Genomic Data Commons (GDC) data portal (https://portal.gdc.cancer.gov/ (accessed on 28 October 2024)).

## Supplementary Materials

The following supporting information can be downloaded at: https://www.mdpi.com/article/10.3390/ijms252312929/s1.
