# Supplementary material for: Integrated Analysis of Single-Cell and Bulk RNA Sequencing Reveals HSD3B7 as a Prognostic Biomarker and Potential Therapeutic Target in ccRCC
Source: Int J Mol Sci. 2024 Dec 1;25(23):12929. doi: 10.3390/ijms252312929 (PMC11641532; doi:10.3390/ijms252312929)
Supplement: Supplementary file 1 [file ijms-25-12929-s001.zip › Figure legend.pdf]

Figure S1. Expression of HSD3B7 in integrated scRNA-seq analysis. (A) HSD3B7 expression in integrated scRNA-seq datasets (GSE131685, GSE152938, GSE156632, and GSE159115) with cell type annotations. (B) HSD3B7 expression in a focused analysis of Epi_Normal/tumor cells extracted from the integrated scRNA-seq dataset. Each dot represents a cell, each color represents a cell type, and the area of the color block represents the number of cells.

Figure S2. Validation of HSD3B7 knockdown efficiency in 769-P cells at the mRNA level. Relative mRNA levels of HSD3B7 in 769-P cells at 12, 24, 48, 72, and 96 hours post-siRNA transfection, as determined by qRT-PCR.
