# Supplementary figures and images for: Integrated Analysis of Single-Cell and Bulk RNA Sequencing Reveals HSD3B7 as a Prognostic Biomarker and Potential Therapeutic Target in ccRCC
Source: Int J Mol Sci. 2024 Dec 1;25(23):12929. doi: 10.3390/ijms252312929 (PMC11641532; doi:10.3390/ijms252312929)

A

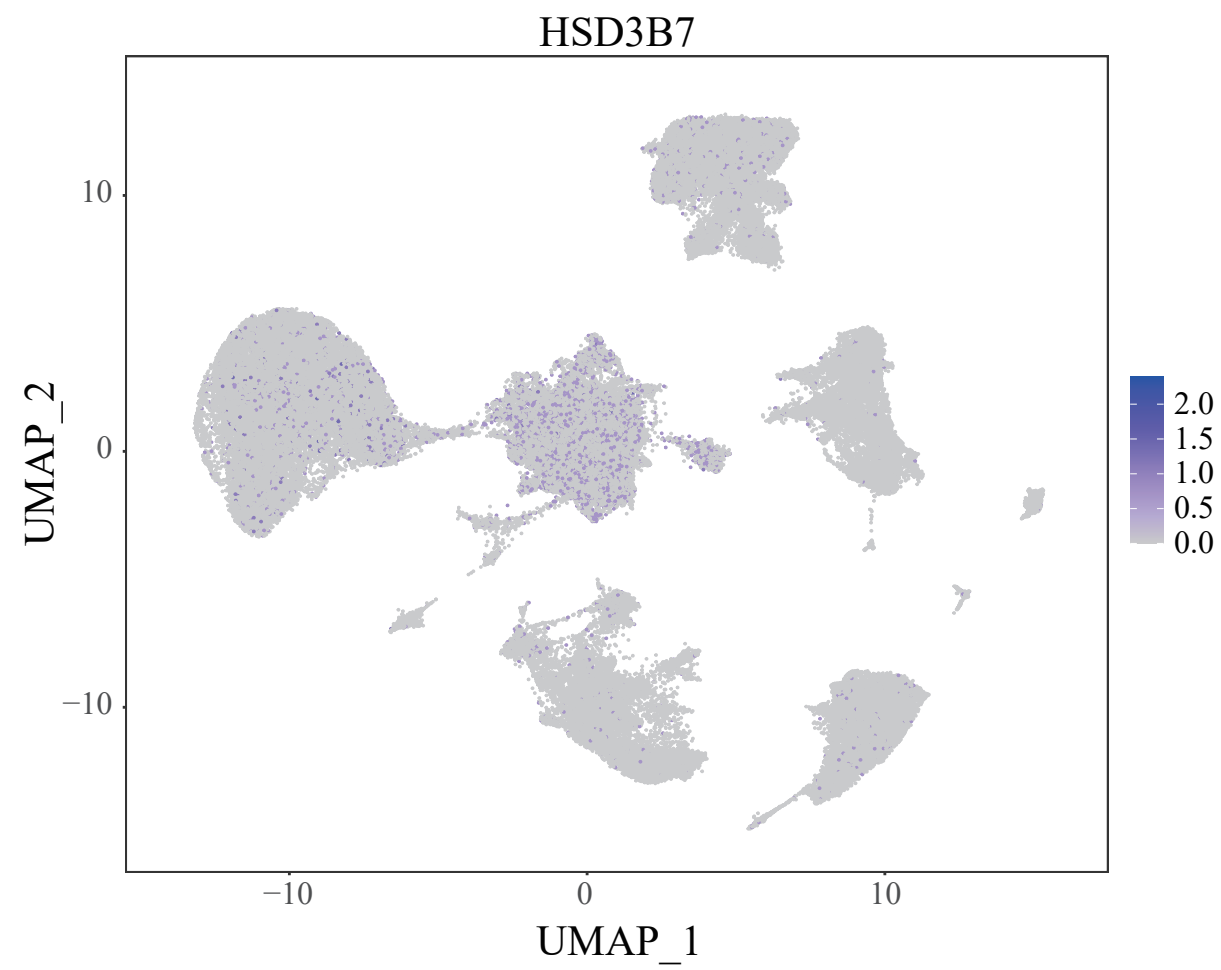

B

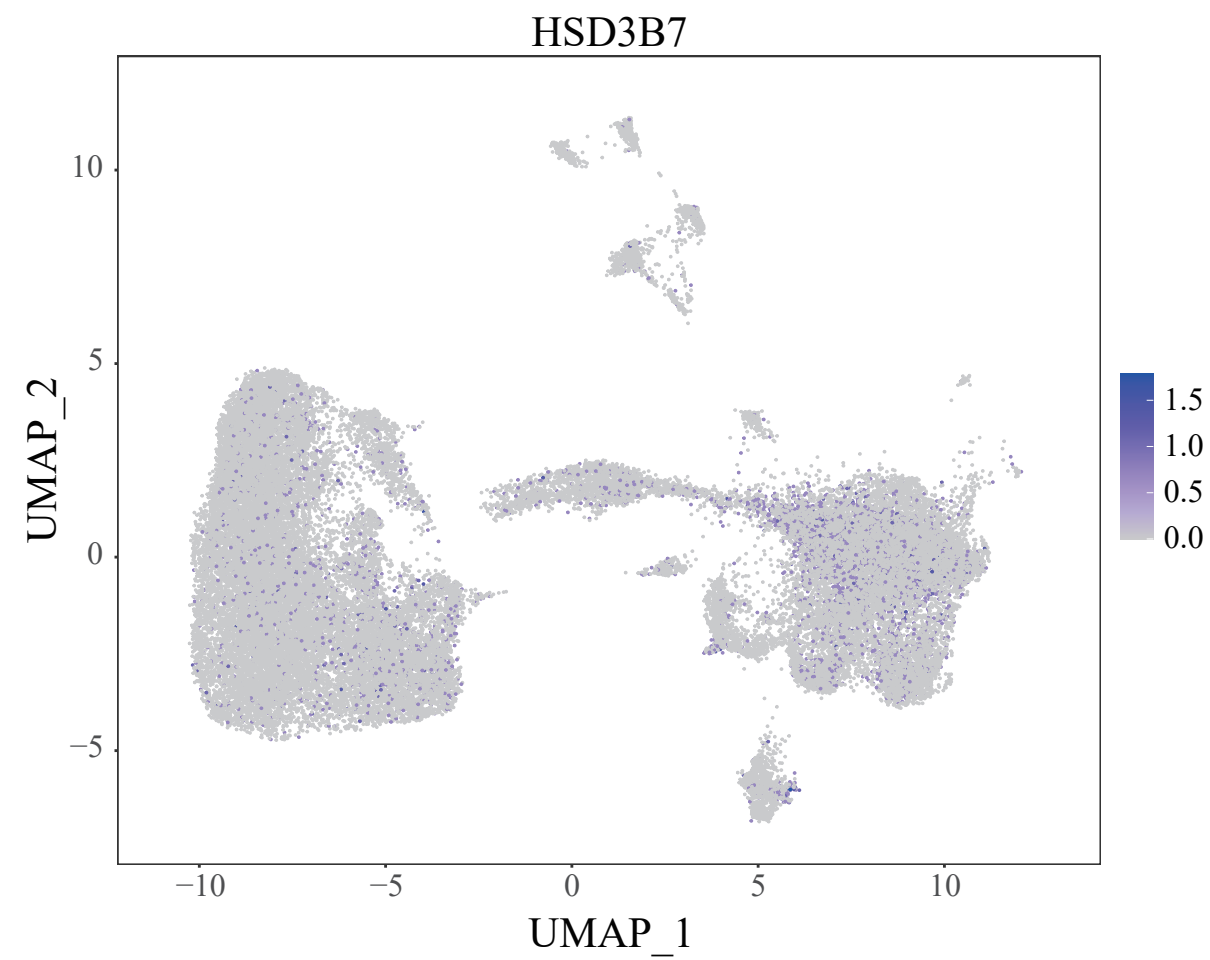

Supplement: Supplementary file 1 [file ijms-25-12929-s001.zip › Figure S1.pdf]

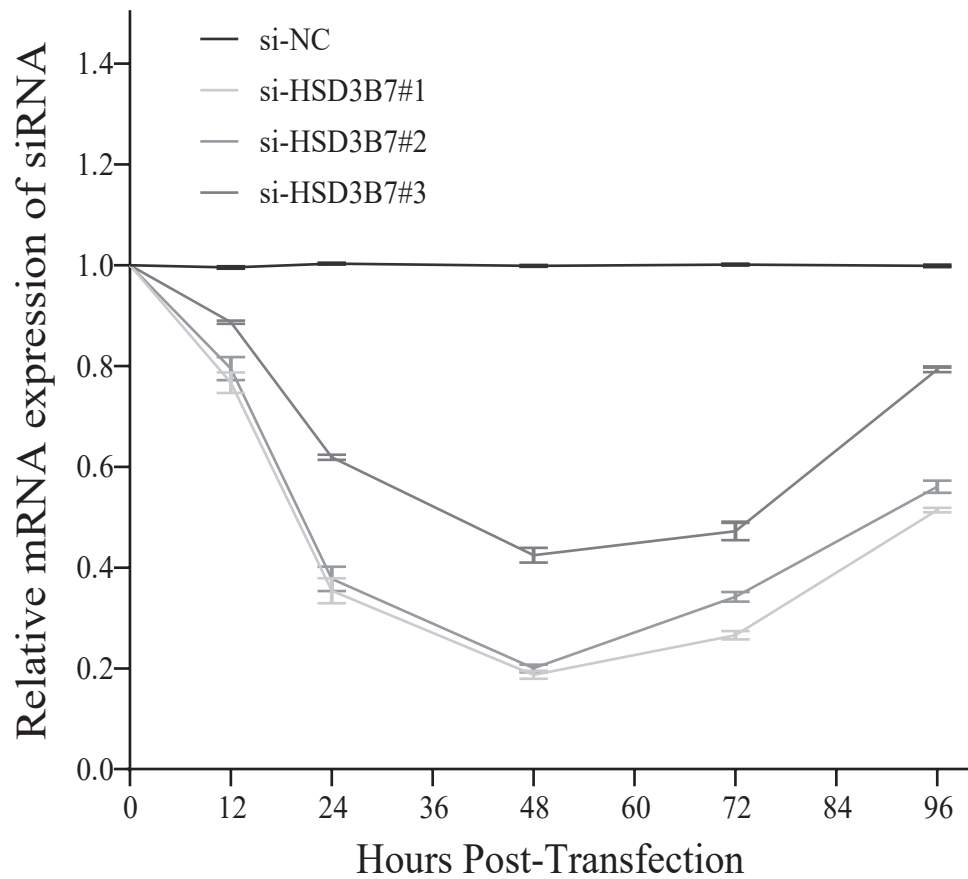

Supplement: Supplementary file 1 [file ijms-25-12929-s001.zip › Figure S2.pdf]
